# Proteome profiling reveals changes in energy metabolism, transport and antioxidation during drought stress in *Nostoc flagelliforme*

**DOI:** 10.1186/s12870-022-03542-8

**Published:** 2022-04-01

**Authors:** Xiaoxu Li, Miaomiao Ding, Meng Wang, Shujuan Yang, Xiaorong Ma, Jinhong Hu, Fan Song, Lingxia Wang, Wenyu Liang

**Affiliations:** College of Life Sciences, Ningxia University, Yinchuan, 750021 China

**Keywords:** Drought stress, Proteome, Differential expression, Functional analysis, *Nostoc flagelliforme*

## Abstract

**Background:**

Drought is an important abiotic stress that constrains the growth of many species. Despite extensive study in model organisms, the underlying mechanisms of drought tolerance in *Nostoc flagelliforme* remain elusive.

**Results:**

We characterized the drought adaptation of *N. flagelliforme* by a combination of proteomics and qRT-PCR. A total of 351 differentially expressed proteins involved in drought stress adaptation were identified. It was found that the expression of several nutrient influx transporters was increased, including molybdate ABC transporter substrate binding protein (modA), sulfate ABC transporter substrate-binding protein (sbp) and nitrate ABC transporter (ntrB), while that of efflux transporters for toxic substances was also increased, including arsenic transporting ATPase (ArsA), potassium transporter (TrkA) and iron ABC transporter substrate-binding protein (VacB). Additionally, photosynthetic components were reduced while sugars built up during drought stress. Non-enzymatic antioxidants, orange carotenoid protein (OCP) homologs, cytochrome P450 (CYP450), proline (Pro) and ascorbic acid (AsA) were all altered during drought stress and may play important roles in scavenging reactive oxygen species (ROS).

**Conclusion:**

In this study, *N. flagelliforme* may regulates its adaptation to drought stress through the changes of protein expression in photosynthesis, energy metabolism, transport, protein synthesis and degradation and antioxidation.

**Highlights:**

• A total of 351 DEPs involved in adaptation to drought stress were identified.

• Changes in the expression of six OCP homologs were found in response to drought stress.

• Differential expression of transporters played an important role in drought stress adaptation.

• Most PSII proteins were downregulated, while PSI proteins were unchanged in response to drought stress.

• Sugar metabolism was upregulated in response to drought stress.

**Supplementary Information:**

The online version contains supplementary material available at 10.1186/s12870-022-03542-8.

## Background

*Nostoc flagelliforme* is a terrestrial nitrogen-fixing cyanobacteria that plays important ecological roles and has high economic value. It is considered to be a local ecosystem pioneer and plays a crucial role in the carbon and nitrogen balance in native habitat. It is also involved in the formation of biological desert soil crusts, which will increase soil organic matter and promote nutrients recycling, thus contributing to species diversity and ecosystem stability [1]. It is primarily located in dry and windy areas with relatively poor species diversity with low rainfall and relative humidity. Due to its challenging natural environment, *N. flagelliforme* typically faces periodic rehydration and dehydration changes [2, 3]. It can survive for decades under extremely dry conditions, and quickly recover its physiological and metabolic state after reabsorbing water [4]. Therefore, *N. flagelliforme* colonies grown under natural conditions are ideal research material for studying drought tolerance mechanisms.

A significant amount of research has been conducted investigating dehydration-responsive proteins and understanding the molecular mechanisms underlying their regulation [5–8]. It has been reported that hairy structures and extracellular polysaccharide sheaths are related to drought tolerance in *N. flagelliforme* [9]. Additionally, the number and volume of vacuoles have been reported to be reduced during dehydration [4]. Transcriptomic studies have also revealed that the expression of genes involved in photosynthesis, starch and sucrose metabolism are correlated during response to drought, with sucrose and trehalose playing an important role in osmotic regulation, stress protection and reactive oxygen scavenging [9]. In addition, a comparative transcriptomic and physiological analysis has shown that dehydration increases the content of exopolysaccharides and the level of reactive oxygen species (ROS) in *N. flagelliforme* [10].

Proteomics studies have improved our understanding of the molecular mechanisms involved in plant stress tolerance [11]. Thus far, the proteomes of several cyanobacteria have also been studied during response to different stresses [12–14], including *Arthrospira platensis* and *Synechocystis* sp. PCC 6803. *Synechocystis* sp. PCC 6803 was subjected to variable light conditions and found to reduce phycobilisome (PBS) antennas and increase photosystem II (PSII) repair mechanisms in orange-red light conditions. Furthermore, proteomics studies on *N. flagelliforme* have found that photosynthesis, antioxidant systems and energy metabolism were affected by different stresses in different ways [1, 4, 15–18]. Transporters have been found to be involved in response to the rehydration stress by *N. flagelliforme* [1]. In addition, carotenoid-binding proteins have also been found to be regulated in response to rehydration and dehydration treatments [7]. These studies provide a basis for exploring the mechanisms underlying the response of *N. flagelliforme* to different stresses and may contribute to the elucidation of the specific metabolic and regulatory mechanisms of *N. flagelliforme*.

To clarify the key events related to drought stress from a protein expression level, we utilized the label-free [19] and LC-MS-MS technologies, as well as genomic databases for *N. flagelliforme*. We also combined parallel response monitoring (PRM) and qRT-PCR to better understand the regulatory mechanisms of drought stress-related proteins in *N. flagelliforme*.

## Results

### Overview of proteome changes and differentially expressed proteins

**Fig. 1:**
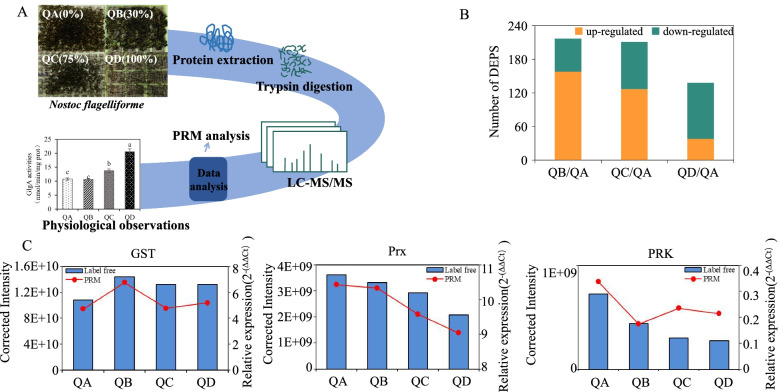
Profiling and validation of the proteome results of *N. flagelliforme*. **A** General overview of the steps involved; **B** Quantitative statistics for differentially expressed proteins (DEPs); **C**. PRM analysis of GST, Prx and PRK under drought stress in *N. flagelliforme.* The values are represented as means ± SD (*n* = 3) in the study (*P* < 0.05)

An overview of the experimental process is summarized in Fig. 1A. SDS-PAGE revealed that the protein bands of the four groups of samples were clear, indicating that the protein quality met the requirements of the downstream experiments (Supplementary Fig. S1). A total of 1617 proteins of *N. flagelliforme* were identified by label-free technology and LC-MS-MS. The total number of proteins present in all four treatments was 1395, and the specific proteins in QA, QB, QC and QD treatments were 8, 7, 8 and 26, respectively (Supplementary Fig. S2). The proteins with a change > 1.2-fold or < 0.83-fold and a *P*-value < 0.05 were considered differentially accumulated proteins [11]. In total, 158 proteins were upregulated and 59 proteins were downregulated at a water loss rate of 30% of colonies compared to a water loss rate of 0% (QB/QA). Additionally, 127 proteins were upregulated and 84 proteins were downregulated at a water loss rate of 75% compared with a water loss rate of 0% (QC/QA), while 38 proteins were upregulated and 100 proteins were downregulated at a water loss rate of 100% compared with a water loss rate of 0% (QD/QA) (Fig. 1B). As drought stress increased, the protein response weakened, which was reflected in the changes in the number of differential proteins.

In order to further verify the expression trend of differentially expressed proteins and the reliability of the label-free quantitative proteomics results, glutathione-S-transferase (GST), peroxiredoxins (Prx) and ribulokinase (PRK) were verified by PRM. This analysis confirmed that the expression trends of GST and Prx were consistent with the proteome results in response to drought stress, and the expression trend of PRK was mostly consistent with the proteome results in response to drought stress (Fig. 1C). These data indicated that the label-free system results were reliable and suitable for further analysis.

### FGO and KEGG enrichment analysis

**Fig. 2:**
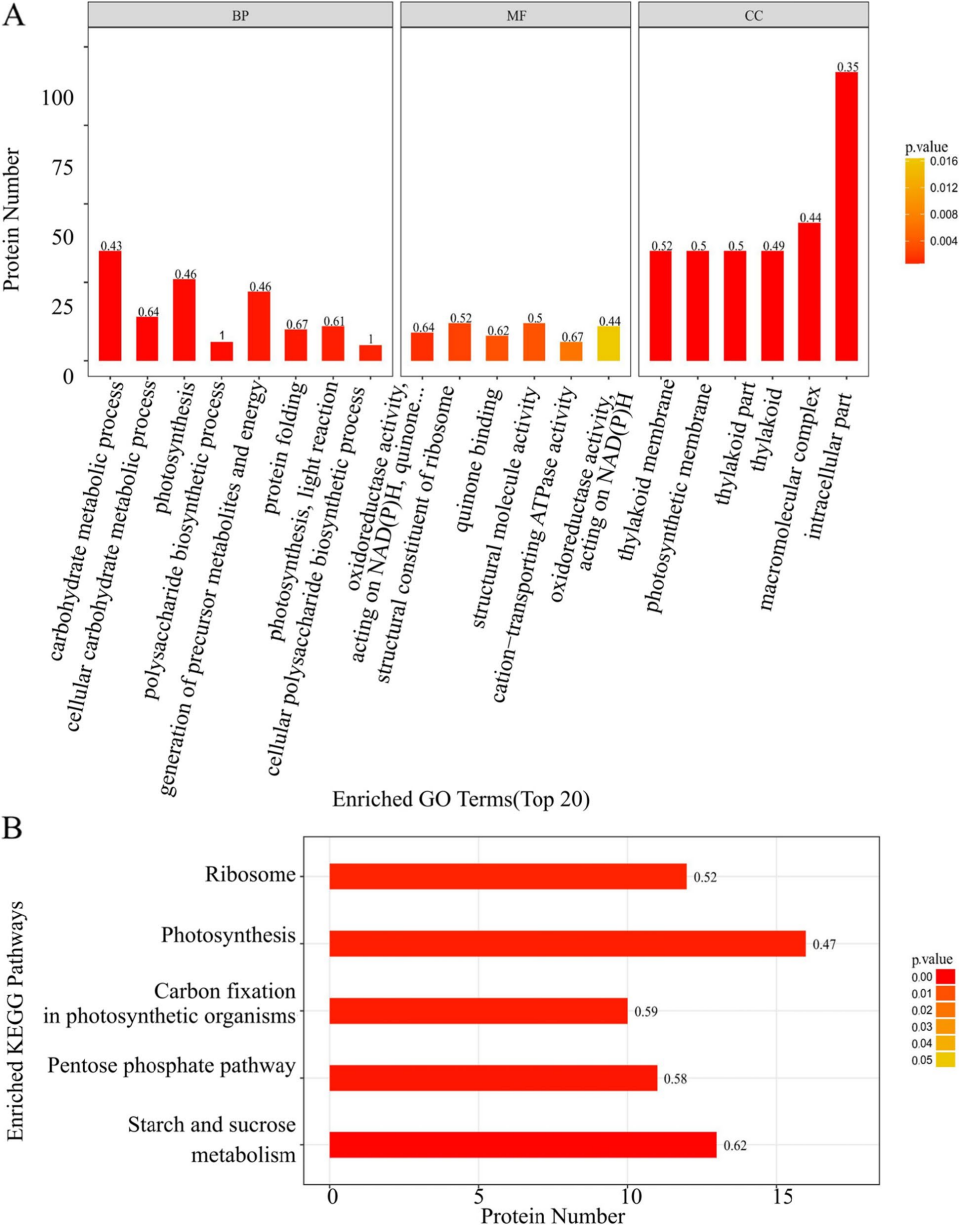
GO and KEGG pathway analyses of differentially expressed proteins in *N. flagelliforme* under drought stress. **A** GO analysis; **B** KEGG pathway analysis

The differentially expressed proteins screened by one-way ANOVA were next analyzed for GO functional enrichment. Eight types of biological processes (BP) were enriched in this set, including important biological processes such as carbohydrate metabolic process, photosynthesis and polysaccharide biosynthetic process, indicating that *N. flagelliforme* may adapt to drought through polysaccharide synthesis. Six molecular functions (MF), including oxidoreductase activity acting on NAD (P) H, ribosome structure and cation transport ATP activity, were enriched and six cellular components (CC), including thylakoid membrane, photosynthetic membrane and intracellular part were enriched (Fig. 2A). KEGG pathway enrichment analysis of differentially expressed proteins showed significant changes in ribosome metabolism, photosynthesis, carbon fixation, pentose phosphate pathway and starch and sucrose metabolism (*P* < 0.05) (Fig. 2B). It was also found that transporters and stress responsive proteins were enriched (Supplementary Table S1), indicating that these pathways may play an important role in regulating *N. flagelliforme* response to drought stress.

### Protein expression trend of *N. flagelliforme* under drought stress

**Fig. 3:**
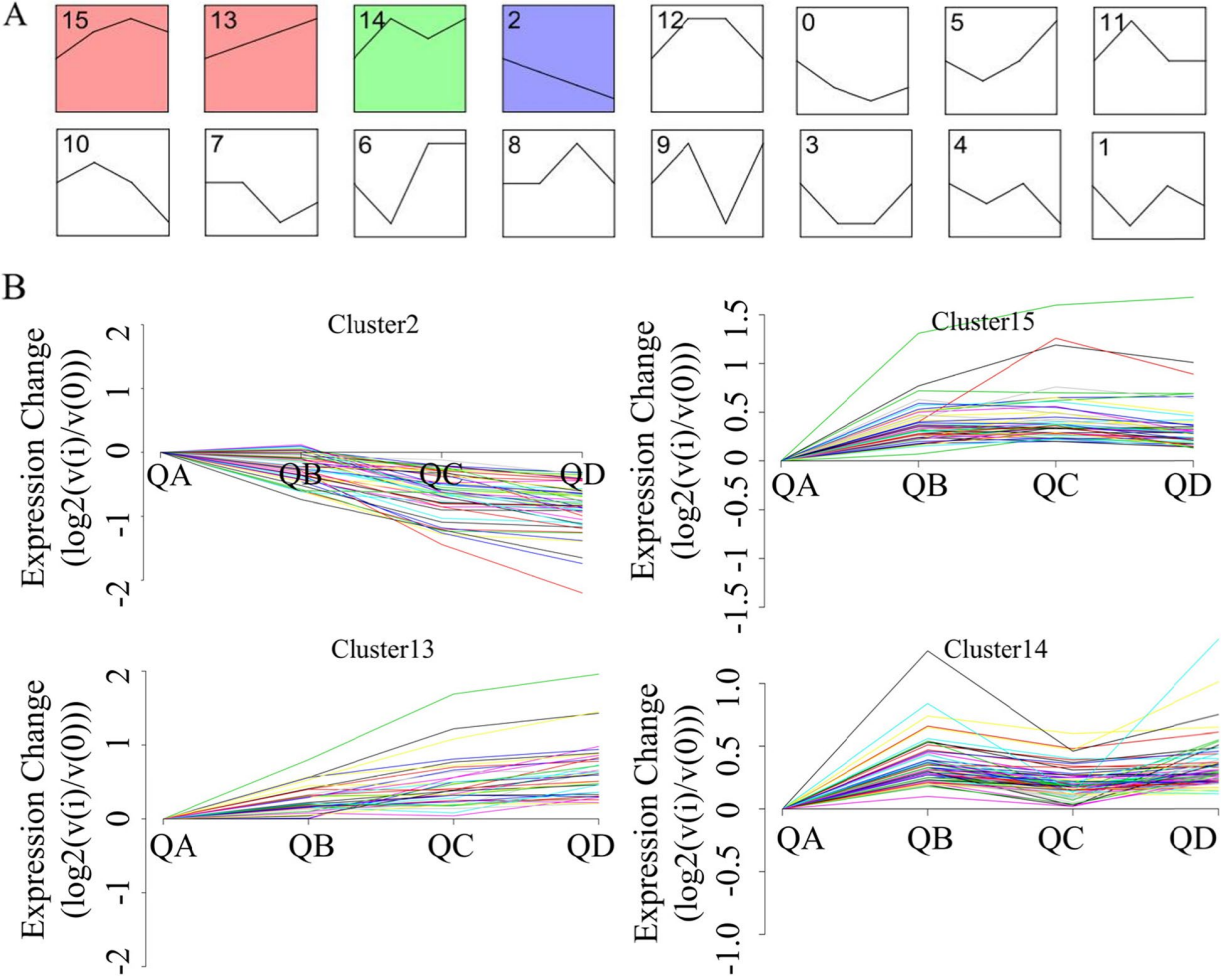
Protein expression trends of *N. flagelliforme* under drought stress. **A** All expression trends; **B** Trends of differential protein expression of *N. flagelliforme* under drought stress. Significant trends were found in Cluster 2, Cluster 15, Cluster 13 and Cluster 14

The changes in protein expression with increasing drought severity were obvious via a cluster analysis. A total of 351 differentially expressed proteins were identified by one-way ANOVA, which clustered into 16 expression patterns (Fig. 3A). There were 214 differentially expressed proteins in four largest differential expression patterns (*P* < 0.05), including Cluster 15, Cluster 13, Cluster 14 and Cluster 2 (Fig. 3B and Supplementary Table S2). The expression trends of Cluster 13 and Cluster 15 were similar, with each cluster containing 52 and 53 differential proteins, respectively. Cluster 13 mostly contained proteins associated with starch and sucrose metabolism and protein synthesis and degradation, while Cluster 15 contained genes involved in protein synthesis and degradation and oxidative phosphorylation, and the response regulator (REC) protein and multiple response regulator transcription factors, increased during drought stress (Supplementary Table S1).

Cluster 2 contained 52 differential proteins, all of which decreased gradually in response to drought stress. Many proteins in this cluster were involved in detoxification and antioxidant activity, photosynthesis and protein synthesis and degradation. Cluster 14 contained 70 differential proteins, all of which rapidly increased at QB, then decreased at QC, followed by an increase at QD. This cluster mainly contained proteins associated with starch and sucrose metabolism, purine pyrimidine metabolism and photosynthetic photoreaction.

### Protein interaction network of *N. flagelliforme* under drought stress

**Fig. 4:**
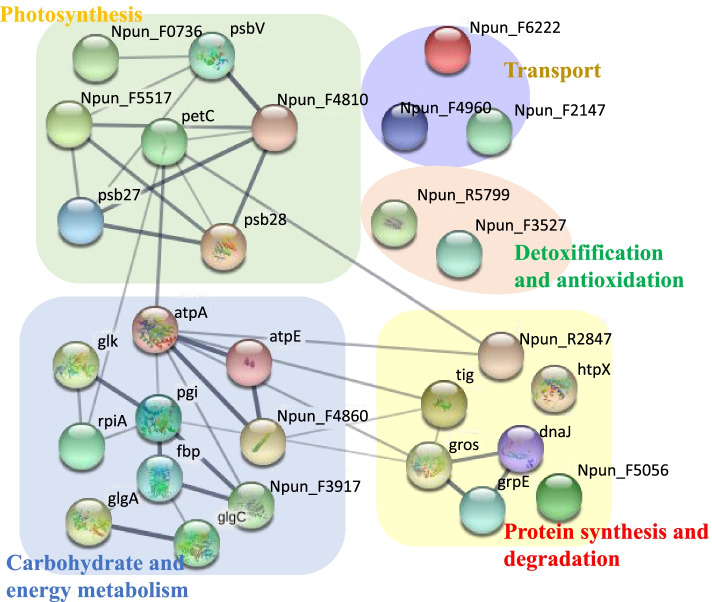
The protein interaction network of *N. flagelliforme* under drought stress. Npun_F5517 is PsbP, Npun_F4810 is PsbO, Npun_R2847 is M16, Npun_F4960 is TrkA, Npun_F3527 is GST, Npun_F2147 is ArsA, htpX is M48, Npun_F6222 is VacB, Npun_F3917 is FBP, Npun_F5056 is S41 and Npun_F3917 is GlpX

Based on the results obtained from GO and KEGG pathway analyses, an interaction network was constructed around biological processes, including photosynthesis, carbon fixation, starch and sucrose metabolism, protein synthesis and degradation, detoxification and antioxidation, transport. Fructose-bisphosphate aldolase (FBP) was higher in glucose metabolism, and groS connectivity was higher in protein synthesis and degradation pathways. These highly connected proteins may play important roles in the adaptation of *N. flagelliforme* to drought stress (Fig. 4), and were found to undergo significant changes during drought stress.

### Physiological observations

**Fig. 5:**
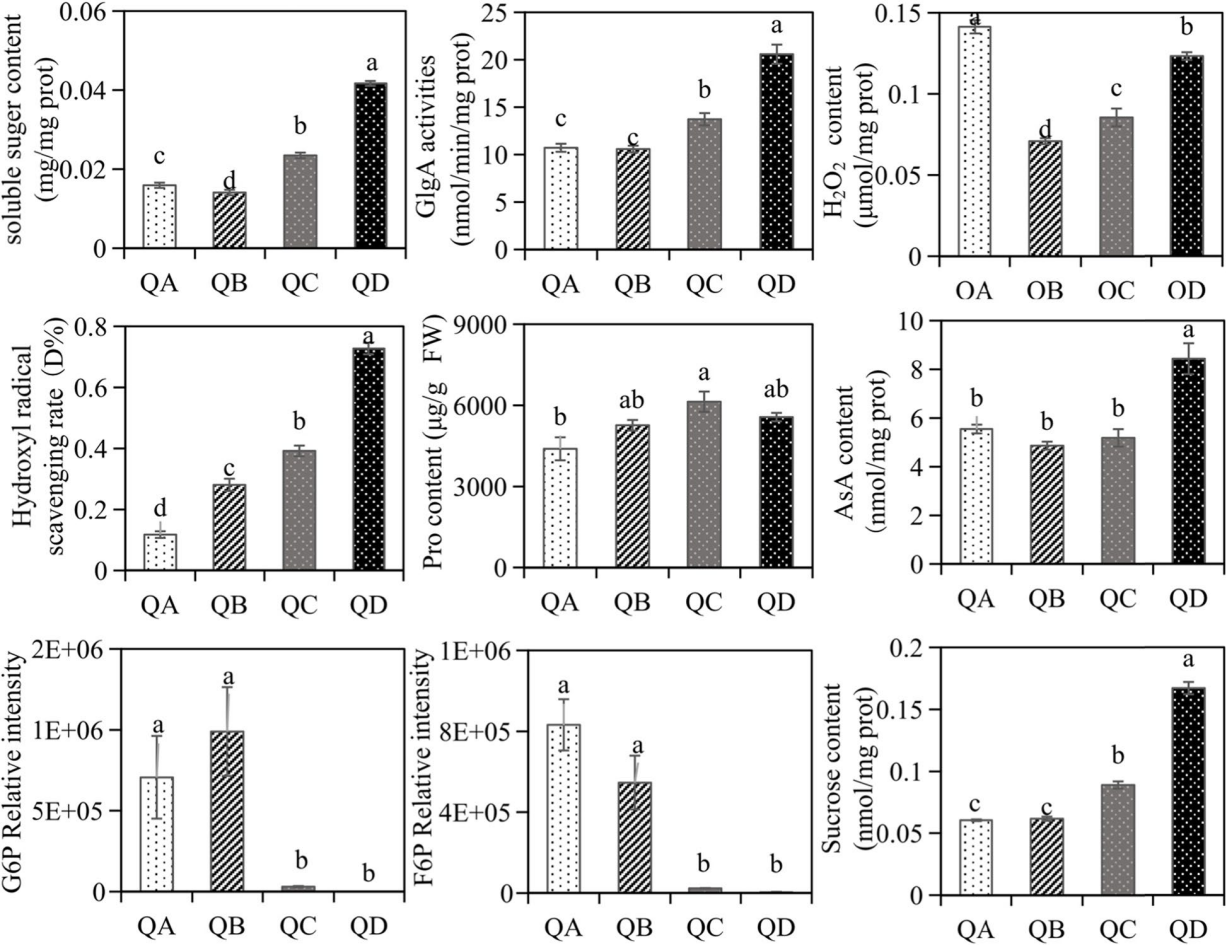
Changes in the metabolite contents and key enzyme activities of *N. flagelliforme* under drought stress. Different letters on each bar indicate significantly different values (*P* < 0.05). The values are presented as means ± standard deviation (*n =* 3) (*P* < 0.05)

The contents of soluble sugar increased by 47.3 and 161.7% at QC and QD when compared with the control (QA), respectively. Additionally, Pro increased by 39.6% at QC, while AsA increased by 51.8% at QD. The hydroxyl radical scavenging rate was sevenfold higher at QD compared to QA. H_2_O_2_ significantly decreased compared with QA, while the activity of GlgA increased by 28.1 and 92.1% at QC and QD. Both the contents of glucose-6-Phosphate (G6P) and fructose-6-phosphate (F6P) decreased at QC and QD, the contents of sucrose increased at QC and QD (Fig. 5).

### qRT-PCR analysis

**Fig. 6:**
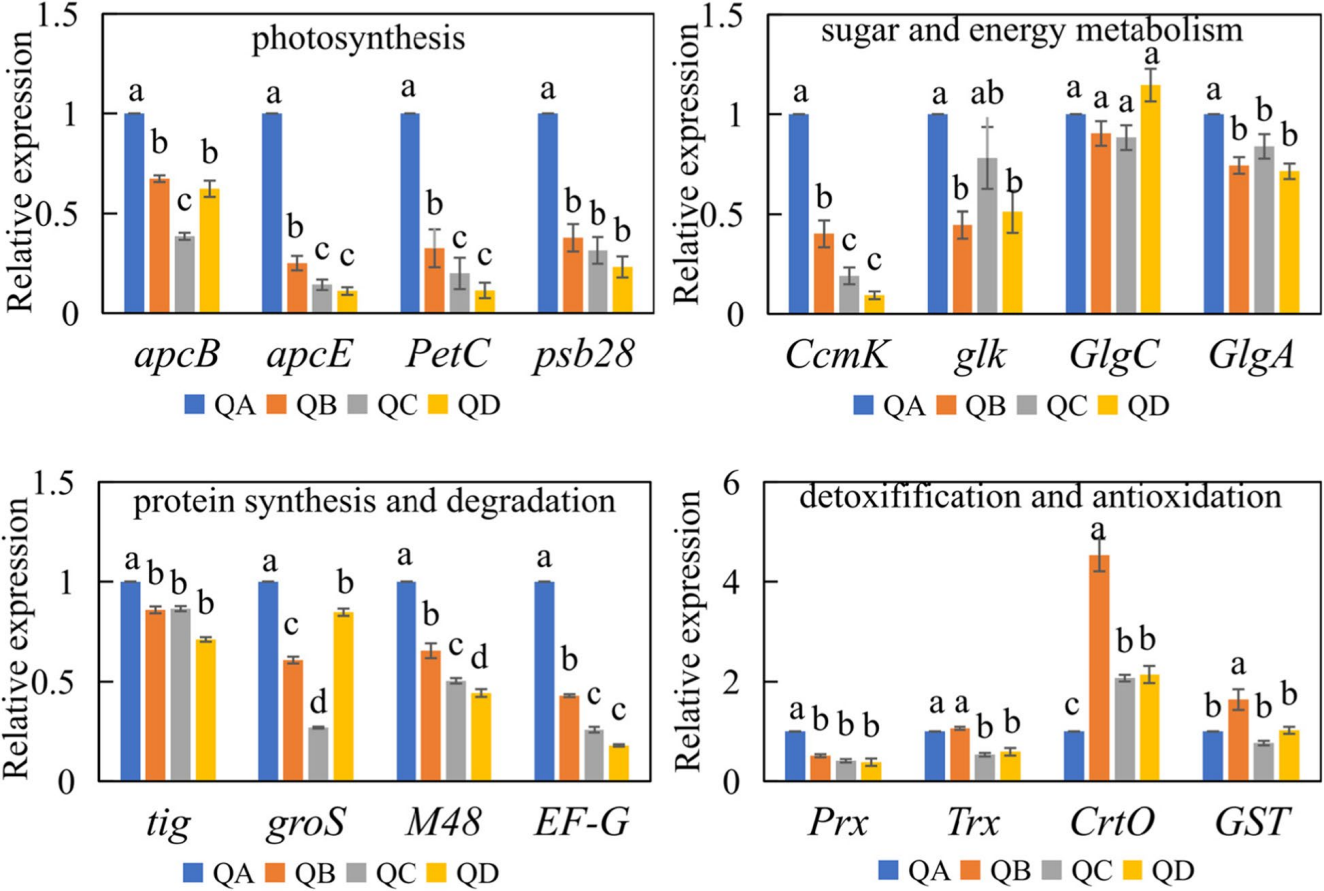
Gene expression under different drought stress conditions. Values (means ± SD) were determined with three replicates. The values are presented as means ± SD (*n =* 3) (*P* < 0.05)

In photosynthesis, the expression levels of photosystem I reaction center subunit X (*apcE*), *psb28*, cytochrome b6-f complex iron-sulfur subunit (*petC*) and allophycocyanin subunit beta (*apcB*) decreased in response to drought stress. The most pronounced decrease was observed for *petC*, which decreased tenfold at QD compared with QA. In the sugar and energy metabolism category, *GlgC* showed no significant changes, while *GlgA*, *Ccmk* and *glk* decreased by twofold to tenfold during dehydration. In the protein synthesis and degradation category, the expression levels of *tig*, *M48*, *EF-G* and *groS* decreased in response to drought stress. In the detoxification and antioxidation category, *Prx* and *Trx* decreased in response to drought stress, while *GST* increased at QB, and *CrtO* levels rose by two to four times at different drought stages (Fig. 6).

## Discussion

### Proteins involved in transport in *N. flagelliforme*

In the phototrophic-heterotrophic system, nutrients are obtained via active membrane transport systems [1]. Additionally, transporters play an important role in efflux of heavy metal ions and stress adaptation [20], making both influx and efflux critical components of the transport system [21]. The substrates of the inward transport system are often nutrients, such as phosphates, sugars and inorganic ions. In this study, multiple phosphate ABC transporter substrate binding proteins (PstS and PhnD) decreased (Fig. 8, Supplementary Table S1), indicating that intracellular phosphate may be sufficient under drought stress. In *Escherichia coli*, PstS1 and PstS2 have been shown to only work when the environmental phosphate concentration is low [22], which is consistent with the changes in phosphate transporters that we observed. The levels of multiple transporters also increased (Fig. 8), including modA, sbp and ntrB, indicating that these transporters provided vital nutrients for *N. flagelliforme* under drought stress. The sugar ABC transporter substrate binding protein (SuaB) is specific for trehalose/maltose and we found that it increased in response to drought stress in *N. flagelliforme* (Fig. 8). The acquisition of sugar has previously been shown to increase under drought stress [23], which is consistent with our results. In addition to competing for limited nutrients, toxic substances in the cell also need to be actively excreted. The transport system related to the efflux of heavy metal ions has been shown to be involved in detoxification and maintaining osmotic pressure in *Mycobacterium tuberculosis* [24]*.* Our results showed that the levels of ArsA, TrkA and VacB significantly increased under different drought stresses (Fig. 8), which may indicated that heavy metals may need to be excreted as part of drought stress adaptation. Overall, transporters appear to be critical for the response of *N. flagelliforme* to drought stress.

### Effects of drought stress on the energy metabolism of *N. flagelliforme*

Photosynthesis is generally believed to be sensitive to drought stress [4]. Six proteins of PSII decreased in response to drought stress (Fig. 8). Psb28 has been shown to protect RC47 assembly intermediates of PSII, its absence impaired PSII recovery after photodamage at high temperature and high-light conditions [25]. The protein and transcript levels of psb28 decreased significantly under drought stress (Fig. 6), suggesting that PSII turnover may be affected. Most proteins of PSI did not change significantly (Supplementary Table S1) and the ultrastructure of the colony cells remained intact under dehydration (Supplementary Fig. S3). Other studies have indicated that photosynthetic activity reduces in dehydrated colonies [4]. PetC and ferredoxin-NADP^+^ reductase (FNR) have been shown to participate in photosynthetic electron transport [26, 27], the levels of these proteins decreased (Fig. 8). The *petC* gene (Fig. 6) and the *petH* gene (*FNR*) [28] were also decreased. The downregulation of *FNR* could increase circulating electron flow and therefore resistance to drought stress [29]. Similarly, *FNR* was decreased in wheat and *Populus cathayana* in response to drought stress [30, 31]. Taken together, unchanged PSI proteins and down regulation of other proteins revealed that the photosynthesis system maintains a lower functionality and ability of electron transfer, which prevents damage to the photosynthetic structures during drought stress.

Previous studies have shown that drought-responsive proteins often participate in energy metabolism [32]. Carbohydrates are one of the most abundant metabolites in plants and play an important role as a source of energy in response to abiotic stresses. In this study, several proteins (FBP, GlpX, PGI) involved in energy metabolism also increased in response to drought stress (Fig. 8). The enhanced expression of these proteins may promote the rate of glycolysis to produce F6P, which is the main material for the synthesis of sucrose and polysaccharides, which may help in response to dehydration, as extracellular polysaccharides are important in protecting the cell membrane [16]. The determination of the content of carbon metabolites in the glycolytic pathway (EMP) of *N. flagelliforme* found that the content of intermediate carbon metabolites F6P and G6P with a water loss rate of 0% was significantly higher than that of other treatments (Fig. 5). These results all indicate that the EMP pathway is an important energy supply pathway in response to drought stress.

Cyanobacteria have a unique carbon concentration mechanism (CCM), which can promote the carboxylation of ribulose-1,5-bisphosphate carboxylase/oxygenase (RuBisCO). RuBisCO is the key enzyme of CO_2_ assimilation in photosynthesis. The expression of ribulose-1- diphosphate carboxylase small subunit (rbcS) and PRK decreased in response to drought stress (Supplementary Table S1), indicating that carbon fixation may be inhibited or maintained at a low level under drought stress.

Glucose-1-adenosyl transferase (GlgC) and GlgA are enzymes for glycogen metabolism. The expression and activities of GlgA were increased in response to drought stress (Figs. 5 and 8), GlgC was also significantly increased (Fig. 8). GlgC and GlgA can catalyze G1P to produce glycogen. Furthermore, sucrose plays an important role in regulating the osmotic potential of cells and stabilising their structural components [16]. Sucrose synthase (SS), a key enzyme in sucrose metabolism, was significantly increased (Fig. 8). Sucrose accumulation induced by salt stress was reported [33]. Therefore, sucrose (Fig. 5) and glycogen [34] were accumulated in response to drought stress, due to increased expression of GlgC and GlgA, SS, and increased activity of GlgA, rather than changes in transcript levels (Fig. 6). Furthermore, the contents of soluble sugar increased in response to drought stress in *N. flagelliforme* (Fig. 5). In short, *N. flagelliforme* may maintain a high content of sugar under drought stress, which helps alleviate drought-induced damage to *N. flagelliforme* cells.

### Effects of drought stress on detoxification and antioxidation in *N. flagelliforme*

Photoautotrophic cyanobacteria and higher plants can produce harmful ROS during abiotic stress, which can cause lipid peroxidation and cell damage [35, 36]. GST catalyzes the binding of electrophilic groups of endogenous or foreign harmful substances with the sulfhydryl groups of glutathione to form non-toxic derivatives [37]. GST significantly increased during drought stress in *N. flagelliforme* at both the transcript level [28] and protein level (Supplementary Table S1). Several proteins involved in the removal of H_2_O_2_ decreased in response to drought stress in *N. flagelliforme*, including Prx, Trx and cytochrome C peroxidase (CCP). Prx proteins are a large family of peroxidases [38], which main involved in the cytokine signal cascade and other functions by regulating the intracellular concentration of H_2_O_2_, and different types of Prx perform different functions [39]. In this study, Prx significantly decreased under different drought stresses (Supplementary Table S1). At the transcript level, *Prx* and *Trx* genes also decreased (Fig. 8). Speculating the low expression of two Prx may be the result of reduced overall metabolic activity of *N. flagelliforme* cells, or the clearance of H_2_O_2_ may be through other pathways, and other studies have also shown that Trx and Prx decreased in response to salt treatment [40]. In this study, cytochrome P450 (CYP450) increased in response to drought stress (Supplementary Table S1), while H_2_O_2_ decreased (Fig. 5). This is consistent with other studies that found an increase in some CYP450 members in cotton during drought stress [41]. Furthermore, overexpression of CYP450 gene conferred enhanced resistance to salt stress via decreased production of H_2_O_2_ accumulation in *Arabidopsis thaliana* [42]. These results suggest that *N. flagelliforme* primarily relies on CYP450 rather than Trx, Prx or CCP to scavenge H_2_O_2_ during drought stress.

**Fig. 7:**
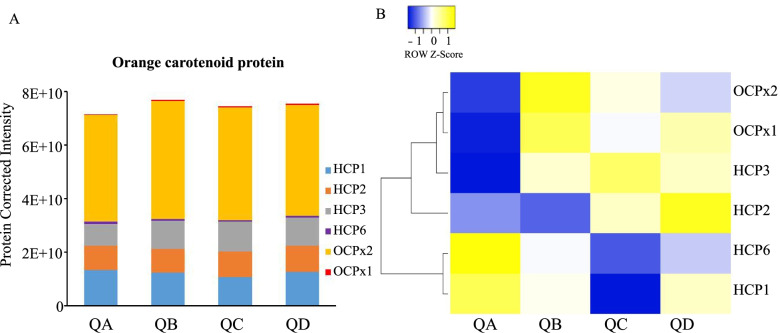
Expression of orange carotenoid proteins (OCPs) in *N. flagelliforme* under drought stress. **A**. Comparison of the abundance of OCPs; **B**. Visualization of OCPs. The proteins that increased and decreased in abundance are indicated in yellow and blue, respectively

Carotenoids can quench singlet oxygen [43], with ketocarotene contributing to this process significantly in cyanobacteria. The protein level of beta-carotene ketolase (CrtO), which is involved in the synthesis of ketocarotene [44], increased slightly in *N. flagelliforme* under drought stress, but the level of its transcript increased significantly (Fig. 6). This finding indicated that *CrtO* expression increased to induce ketocarotene formation in order to quench singlet oxygen (^1^O_2_) in response to drought stress. Orange carotenoid proteins (OCPs) can also quench ^1^O_2,_ and *ocp* gene were composed of *hcp1*, *hcp2*, *hcp3*, *hcp6, ccp*, *ocpx1* and *ocpx2* [7]. Our data showed that OCPx2 was the main contributor to total OCP protein, and it increased under drought stress (Fig. 7 and Supplementary Table S1). HCP3 also accounted for a large portion of the total protein expression of OCPs, and it significantly increased at QC and QD (Fig. 7), and *hcp3* also increased at the transcript level during dehydration [7], which indicated that OCPx2 and HCP3 may also play important roles in total OCP function. Moreover, the OCPs from desiccated *N. flagelliforme* quench ^1^O_2_ in vitro [7]. Compared with the control, the total OCP expression increased under drought stress (Fig. 7), which may lead to the elimination of ^1^O_2_.

### Effects of drought stress on protein synthesis and degradation in *N. flagelliforme*

Several proteins (groS, tig) involved in the correct folding and efflux of proteins also significantly decreased during drought stress (Supplementary Table S1). The *groS* and *tig* genes decreased compared with the QA (Fig. 6). These results indicated that the correct folding and efflux of proteins may be seriously affected, which may significantly affect cell survival, since protein degradation is important for removing damaged proteins during drought [32]. Several peptidases were found to increase (Supplementary Table S1), which may serve to hydrolyze proteins that have been damaged during drought stress to free their amino acids for reuse. Despite this, the gene expression level of *M48* decreased in response to drought stress (Fig. 6). Extension factor G (EF-G) promotes ribosome movement to the 3′ end of mRNA [45], and it works in concert with ribosomal recycling factor (RRF) to recycle ribosomes, which is important in the synthesis of new proteins [32]. The EF-G increased significantly (Supplementary Table S1), indicating that the amino acids freed up by proteases were likely reused for new protein synthesis.

## Conclusion

**Fig. 8:**
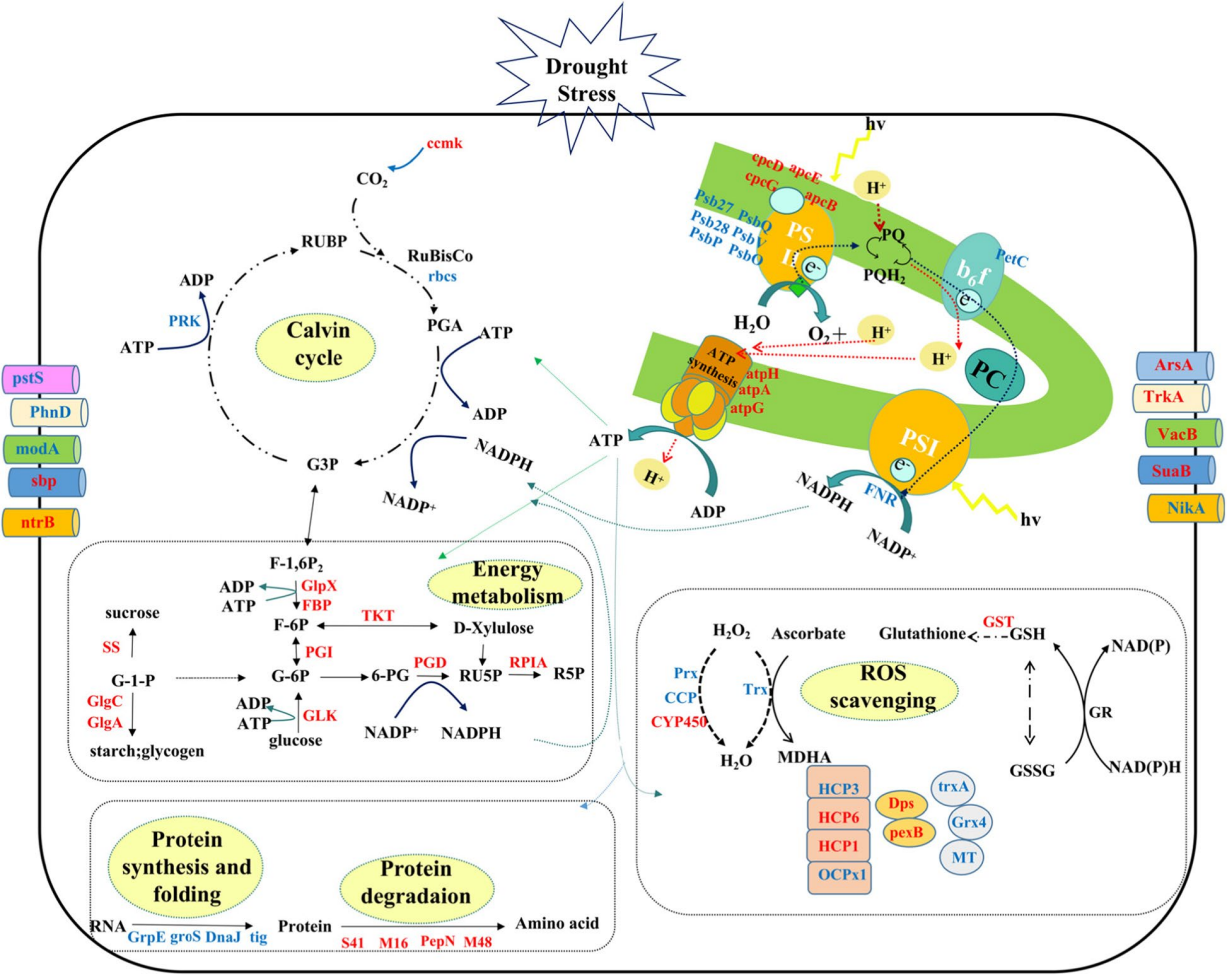
Protein molecular regulation network in *N. flagelliforme* under drought stress. Proteins (drought stress vs. control) that were upregulated are colored red, while proteins (drought stress vs. control) that were downregulated are colored blue

In this study, proteomics combined with qRT-PCR data and metabolites was used to analyse the biology of protein expression regulation of *N. flagelliforme* in response to drought stress. Several differentially expressed transporters were identified. Photosynthetic activity was also maintained at a low level, which was evidenced by a reduction in several proteins associated with this process. Proteins involved in sugar metabolism were upregulated in response to drought stress, while ROS was scavenged by both enzymatic and non-enzymatic systems. Additionally, protein-level changes were seen in six different OCP homologs during drought stress responses. Furthermore, incorrectly folded proteins were hydrolyzed and the resulting amino acids were used to further adapt to drought stress. Therefore, *N. flagelliforme* may regulates its adaptation to drought stress through the changes of protein expression in photosynthesis, energy metabolism, transport, protein synthesis and degradation and antioxidation (Fig. 8). The changes in protein abundance, transcript levels and physiology identified in this study will lay a foundation for further study of the complex molecular processes which occur during *N. flagelliforme* drought stress responses.

## Materials and methods

### Materials

*N. flagelliforme* was provided by Yinchuan Botanical Garden (Yinchuan, China), which was acquired from Helan Mountain in China. It was cultured under conditions designed to mimic the natural environment of *N. flagelliforme*. The culture temperature was 25 ± 2 °C, with a light intensity of 400 μmol/(m^2^·s). QA was sampled when the water loss rate of *N. flagelliforme* was 0% (control group, fully absorbed and held for 4 h), QB was taken when the water loss rate was 30% (fully absorbed and then loses water for about 45 min), QC was taken when the water loss rate was 75% (fully absorbed and then loses water for about 2.5 h) and QD was taken when the water loss rate was 100% (fully absorbed and then loses water for about 64 h). There were at least three biological replicates in each group, and samples were stored at − 80 °C.

### Protein extraction and cleavage, content determination, SDS-PAGE detection and enzymatic hydrolysis

The total proteins of *N. flagelliforme* were extracted by a modified trichloroacetic acid (TCA) / acetone precipitation method [4]. Protein quantitation and SDS-PAGE analysis were conducted as Li et al. described [28]. The protein was visualized by Coomassie brilliant blue staining and decolorization. Each sample was trypsin hydrolyzed by the filter-aided sample preparation (FASP) method [46], and the peptide was desalted by a C18 cartridge. After freeze-drying, the peptide was re-dissolved with 40 μL of 0.1% formic acid solution, and the peptide was quantified by measuring its absorbance at OD_280_.

### LC-MS/MS and data analysis

The HPLC liquid phase system Easy nLC system was used to separate samples. The injection volume is 2 μg peptide. The chromatographic column was balanced with a 95% solution of buffer A (aqueous 0.1% formic acid), with the sample first passed through the trap column (Thermo Scientific Acclaim PepMap100, 100 μm × 2 cm, nanoViper C18), and then separated by the analytical column (Thermo Scientific EASY column, 10 cm, ID 75 μm, 3 μm, C18-A2) with a flow rate of 300 nL/min. The separated samples were analyzed by a Q Exactive mass spectrometer. The detection mode was set to positive ion, with AGC (automatic gain control). Target and maximum IT were 1e6 and 50 ms, respectively. Other settings were used as previously described [47]. The scanning range of the precursor ions was 300 - 1800 m/z; the resolution of MS1 was 70,000 at 200 m/z. The mass charge ratio of peptides and polypeptide fragments was collected according to the following methods: 20 fragment maps (MS2 scan, HCD) were acquired after each full scan. MS2 have a resolution of 17,500 at m/z 200. The software MaxQuant (version 1.5.3.17) was used for database searches, while the LFQ (label free quantitation) algorithm was used for quantitative analysis [48]. The P17036_NCBI_ Nostoc_flagelliforme_18909_ 20,171 228 database was used in this study.

### Bioinformatics analysis

Differentially expressed proteins were screened by one-way analysis of variance (ANOVA; *P*-value < 0.05). The target protein set was annotated with GO and KEGG pathways via Blast2GO [47, 49] and KAAS (KEGG Automatic Annotation Server) software, respectively. GO functional enrichment analysis [50] and KEGG pathway enrichment analysis [51] were carried out by Fisher’s exact tests. The protein–protein interaction between the target proteins was searched via the STRING database (http://string-db.org/).

### PRM verification

PRM verification was carried out for selected proteins with important biological functions. Peptide information was imported into the Xcalibur software and set via the PRM method. Original PRM files were analyzed by Skyline version 3.5.0 [52]. Full details are given in Supplementary Text S1.

### Detection of physiological parameters

The contents of soluble sugar, sucrose, AsA, H_2_O_2_ and hydroxyl radical scavenging rate were measured utilizing the plant soluble sugar assay kit, Micro Ascorbic Acid content assay Kit, Hydrogen peroxide assay kit, Hydroxyl free radical removal capacity determination kit from Suzhou Comin Biotechnology Co., Ltd. (Suzhou, China), according to the manufacturer’s instructions. The content of Pro was measured using an HPLC system [53]. The activity of glycogen synthase (GlgA) was measured according to the instructions for the glycogen synthase kit from Suzhou Comin Biotechnology Co. Ltd. (Suzhou, China).

The contents of G6P and F6P were measured using UPLC-MS. Samples were separated using an Agilent 1290 Infinity LC UPLC system. Mass spectrometry was performed using a 5500 QTRAP mass spectrometer (AB SCIEX) in negative ion mode. Multiple reactions monitoring (MRM) scan type was used in the negative scan mode to detect ion pair. Multiquant software was used to extract peak areas and retention times, and standards (Sigma-Aldrich) of G6P and F6P were used to correct retention times for metabolite identification.

### qRT-PCR analysis

The expression levels of transcripts encoding 16 differential proteins associated with drought stress in *N. flagelliforme* were examined via qRT-PCR (Supplementary Table S3). An RNAprep Pure Plant Kit (Polysaccharides & Polyphenolics-rich) was used to extract the total RNA from *N. flagelliforme*. Primer sequences of the genes are shown in Supplementary Table S3. The cDNA synthesis kit Revert Aid Premium Reverse Transcriptase (Thermo Scientific synthesis EP0733) was used for cDNA synthesis, with 16sRNA used as an internal reference gene. At least three biological replicates were used for each sample. The expression levels of 16 genes were quantitated with the 2^-△△Ct^ method [54].

### Data processing and statistical data analysis

Three independent biological replicates were performed, and all measurements are shown as the mean ± SD. One-way ANOVA was used to examine the differences among the treatments (*P* ≤ 0.05).

## Supplementary Information

**Additional file 1.**

**Additional file 2.**

**Additional file 3.**

**Additional file 4.**

## Data Availability

The datasets used and analysed during the current study are available from the corresponding author on reasonable request.

## Abbreviations

ATP: Adenosine triphosphate
HPLC: High performance liquid chromatography
LC-MS/MS: Liquid chromatograph mass spectrometer
qRT-PCR: Quantitative real-time polymerase chain reaction
SDS: Sodium dodecyl sulfate
PAGE: Polyacrylamide gel electrophoresis
GO: Gene Ontology
KEGG: Kyoto Encyclopedia of Genes and Genomes
NADPH: Triphosphopyridine nucleotide
ABC transporters: ATP binding cassette transporters
atpG: F0F1 ATP synthase subunit B
atpA: F0F1 ATP synthase subunit alpha
atpH: ATP synthase subunit delta
modA: Molybdate ABC transporter substrate binding protein
sbp: Sulfate ABC transporter substrate-binding protein
ntrB: Nitrate ABC transporter
NikA: Peptide ABC transporter substrate-binding protein
ArsA: Arsenic transporting ATPase
TrkA: Potassium transporter
VacB: Iron ABC transporter substrate-binding protein
