# Supplementary material for: Proteome profiling reveals changes in energy metabolism, transport and antioxidation during drought stress in Nostoc flagelliforme
Source: BMC Plant Biol. 2022 Apr 1;22:162. doi: 10.1186/s12870-022-03542-8 (PMC8973743; doi:10.1186/s12870-022-03542-8)
Supplement: Supplementary file 1 — Additional file 1. [file 12870_2022_3542_MOESM1_ESM.docx]

**Supporting Information**

**Proteome profiling reveals changes in energy metabolism, transport and antioxidation during drought stress in *Nostoc flagelliforme***

Xiaoxu Li ^a^, Miaomiao Ding ^a^, Meng Wang ^a^, Shujuan Yang ^a^, Xiaorong Ma ^a^, Jinhong Hu ^a^, Fan Song ^a^, Lingxia Wang ^a^, *, Wenyu Liang ^a^, *

^a^ *School of Life Sciences, Ningxia University, Yinchuan 750021, PR China*

Address reprint requests to:

Prof. Wenyu Liang

Address: Helanshan Road, Yinchuan, P.R. China, 750021

Phone number: 86-0951-2062810

Email: liang_wy@nxu.edu.cn; wang_lx0218@163.com

**Running title**: Proteome changes related to drought tolerance

**Text S1**. PRM verification method

About 1 μg peptide was taken from each sample and mixed with 20 fmol standard peptide (PRTC: GISNEGQNASIK, K is a re-labeled amino acid) for detection. HPLC system was used for chromatographic separation. The column was balanced with 95% buffer A (0.1% FA), the sample was injected into the column for gradient separation with a flow rate of 300 nL/min. The liquid phase separation gradient was as follows: 0-2 min, linear gradient of buffer B (0.1% FAAS) from 5% to 10%; 2- 45 min, linear gradient of buffer B from 10% to 30%; 45 -55 min, linear gradient of buffer B from 30% to 100%; 55 -60 min, linear gradient of buffer B maintains 100%. The separated samples were analyzed by Q-Exactive HF mass spectrometer (Thermo Scientific) for PRM mass spectrometry. Analysis time, 60 min; detection mode: positive ion. The scanning range of MS^1^ is 300-1800 m/z, AGC target: 3e6; Maximum IT: 200 ms; and the resolution of the mass spectrometry is 60000. After each primary MS scan of (full MS scan), 20 PRM scans were collected according to Inclusion list (MS^2^ scans), Isolation window: 1.6 Th, MS resolution: 30000 (m/z 200), AGC target: 3e6; Maximum IT: 120 ms; MS^2^ Activation Type: HCD; Normalized collision energy: 27.

**Text S2.** Visualization of *N. flagelliforme* ultrastructure by using transmission electron microscopy.

The samples of *N. flagelliforme* with different treatments (QA was taken when the water loss rate of *N. flagelliforme* was 0% (control group), QB was taken when the water loss rate was 30%, QC was taken when the water loss rate was 75%, and QD was taken when the water loss rate was 100%) were fixed in 2.5% glutaraldehyde for 3 h, and then post-fixed in 1% osmium tetraoxide for 12 h at 4°C. After fixation, the samples were dehydrated in a graded acetone series, and embedded in Epon812 resin. The ultrathin sections (70-80 nm) were made by microtome (Leica UC-6, Wetzlar, Germany). The ultrathin sections were stained with both lead citrate and uranyl acetate, and then viewed by electron microscope (JEM-1200HC).

Fig. S1. SDS-PAGE analysis of *N. flagelliforme* proteins under drought stress. QA-1, QA-2, QA-3; QB-1, QB-2, QB-3; QC-1, QC-2, QC-3; QD-1, QD-2, QD-3 are three repetitions of the samples of water loss rate was 0 %, 30 %, 75 % and 100%, respectively. M, marker. Protein quality analysis was performed.


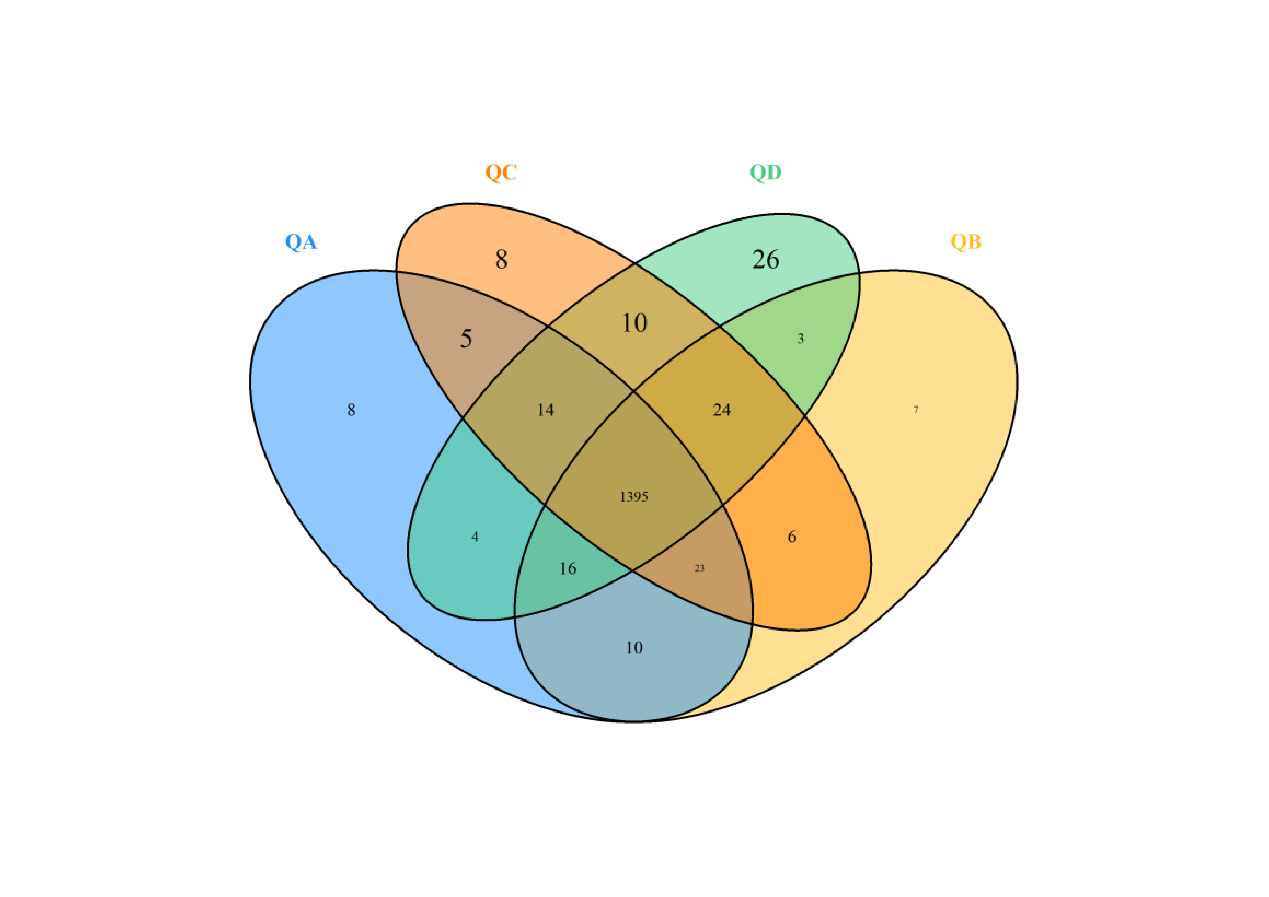


Fig. S2. Venn diagram of identified proteins among the samples of *N. flagelliforme* under drought stress. QA, QB, QC and QD represent the samples of water loss rate was 0 %, 30 %, 75 % and 100%, respectively.

## Fig. S3 Ultrastructure of *N. flagelliforme* under different drought stresses. A, B, C and D ([×](https://jingyan.baidu.com/article/fa4125acb30d8228ac709235.html)5000) represent the samples of water loss rate was 0 %, 30 %, 75 % and 100%, respectively.
